# Reconsideration of the safety and effectiveness of human oocyte cryopreservation

**DOI:** 10.1186/s12958-023-01071-z

**Published:** 2023-02-27

**Authors:** Helen C. K. Kwan

**Affiliations:** 1 Department of Research and Development, KSRS, San Francisco, CA USA; 2 Department of Sciences, Mathematics and Biotechnology, University of California, Berkeley Extension, Berkeley, CA USA

**Keywords:** Aneuploidy, Cryopreservation, Cryoprotective agents, Dimethyl sulfoxide, DNA methylation, Embryonic development, Epigenomics, Fertility preservation, In vitro fertilization, Gene expression profiling, Melatonin, MicroRNAs, Oocytes, Oxidative stress, Transcriptome, Vitrification

## Abstract

Mature oocyte cryopreservation (OC) has become increasingly common since the American Society for Reproductive Medicine declared OC to no longer be experimental. Utilization of the open vitrification protocol has led to a marked improvement in the efficacy of oocyte cryopreservation. However, the safety and effectiveness of this cryopreservation method remain controversial. A previous report stated that among all initiated recipient cycles, the live-birth rate among recipients of all ages was significantly higher when using fresh donor oocytes (FDOs) rather than cryopreserved donor oocytes (CDOs). Confounding patient characteristics were noted as possible causes. OC stands as an acceptable elective medical intervention for preserving fertility in women. To further understand the effects of OC on the live birth rate resulting from fresh versus cryopreserved donor oocytes, reported data from the Society for Assisted Reproductive Technology from 2013 to 2020 were analyzed. The mean of the mean live-birth rate in all ages resulting from FDOs was 49.0% (44.6–53.3%) versus 41.0% (39.1–43.2%) for CDOs (difference, 8.0% [95% confidence interval, 5.35–10.57%], *p* value < 0.001). The lower live-birth rate observed for CDOs versus FDOs has been consistent throughout past decades. While there has been no reported increase in the aneuploidy rate for CDOs compared to FDOs, differences in the nondisjunction separation rate among different chromosomes were described in a recent report. Open vitrification culture medium usually contains high concentrations of cryoprotectants, such as 15% dimethyl sulfoxide (DMSO) and 15% ethylene glycol (EG). Recent studies showed that tissue culture with 0.1% DMSO or 10% EG resulted in deregulation of gene expression, disruption of epigenetic imprints, and accumulation of reactive oxygen species. The addition of melatonin, which can remove reactive oxygen species from vitrification medium, was shown to improve CDOs qualities and functions to conditions similar to those of FDOs; however, there were insufficient data to conclude that melatonin could improve the lower live-birth rate. These factors that affect live birth rates, birth defects, birth weights and developmental health cannot be ignored and perhaps need to be studied again and followed when evaluating the true effectiveness of human oocyte cryopreservation.

## Oocyte banking demand

Oocyte banking or mature oocyte cryopreservation (OC) has become increasingly common in the United States, and perhaps worldwide, since the American Society for Reproductive Medicine (ASRM) and the Society for Assisted Reproductive Technology (SART) announced that OC was no longer considered experimental in late 2012 (Table 1). The ASRM and SART jointly published guidelines for OC practices in early 2013 [1]. The utilization of the open vitrification protocol led to a marked improvement in the efficacy of oocyte cryopreservation [1–5]. Since the ASRM specified that there were no increases in chromosomal abnormalities, birth defects, developmental defects, or differences in birth weight among children born from vitrified oocytes versus (vs.) children conceived from fresh oocytes, the ASRM Practice and Ethics Committees has approved the use of OC in patients receiving therapeutic treatments that could cause infertility. At that time, the Practice Committee declined to recommend OC ‘‘for the sole purpose of circumventing reproductive aging in healthy women’’ [1]. While oocyte banking from autologous users was not recommended to preserve fertility in women, oocyte banking from donors was permissible for oocyte donation.

**Table 1:** Oocyte Banking Instances and Comparison of Mean of Live Birth Rate Percentages from Fresh Versus Cryopreserved Donor Oocyte Cycles Reported to the Society for Assisted Reproductive Technology from 2013–2020. https://www.sartcorsonline.com

	**Total Oocyte Banking Reported (Oocyte Banking reported for Fertility Preservation)**	**Fresh Donor Oocytes Subjected to IVF/ICSI**		**Cryopreserved Donor Oocytes Subjected to IVF/ICSI**		
**Year**		**Number of per initiated recipient cycle from all ages**	**Mean Percentage (%) of recipient initiated cycle resulting in live births (95% CI) from all ages**	**Number of per initiated recipient cycle from all ages**	**Mean Percentage (%) of recipient initiated cycle resulting in live births (95% CI) from all ages**	***P* value**
2013	2227	8,921	49.6% (48.6–50.6)	2,227	43.2% (41.2–45.3)	< 0.001 ^8^
2014	6090 (6090)	6,776	53.3% (52.1–54.4)	2,468	41.0% (39.1–43.0)	
2015	7591 (7591)	5,897	50.5% (49.2–51.8)	2,803	40.8% (39.0–42.7)	
2016	9239 (8336)	4,409	50.6% (49.2–52.1)	2,908	39.4% (37.7–41.2)	
2017	10,955 (9607)	3,146	49.2% (47.4–50.9)	3,013	43.1% (41.3–44.9)	
2018	14,758 (13,041)	2,368	49.4% (47.4–51.4)	3,362	39.1% (37.4–40.7)	
2019	17,805 (15,829)	1,776	44.7% (42.4–47.0)	2,841	40.5% (38.7–42.4)	
2020	18,323 (16,945)	1,277	44.6% (41.9–47.4)	2,541	41.1% (39.2–43.0)	
**Total per initiated recipient cycles from 2013–2020**		34,570		22,163		
**All ages mean of the mean percentages live- birth-rate from** **2013–2020**			49.0% (44.6–53.3)		41.0% (39.1–43.2)	< 0.001

^8^ Kushnir VA, Barad DH, Albertini DF, Darmon SK, Gleicher N. Outcomes of Fresh and Cryopreserved Oocyte Donation. JAMA. 2015;314(6):623–4. 10.1001/jama.2015.7556.

In 2018, the ASRM Ethics Committee found that the use of OC in women attempting to safeguard their reproductive potential for the future was ethically permissible, and the term “planned oocyte cryopreservation (planned OC)” was coined. Planned OC “may enhance women's reproductive autonomy and promote social equality” [6]. “Planned OC”, “OC”, “social egg freezing”, “freezing for nonmedical reasons”, “elective OC”, and “Anticipated Gamete Exhaustion (AGE)” have been used interchangeably to describe the process of preserving oocytes for future fertility. However, there can be reasons for the utilization of planned OC other than maternal age. Common reason for women to choose planned OC include but are not limited to primary ovarian insufficiency or overstimulation, gonadotoxic side effects of cancer or other therapeutic treatments, planned female-to-male transition, the lack of a partner at the time of egg retrieval, a desire for children in response to unanticipated future events such as remarriage, and the elimination of the need for the involvement of third parties such as oocyte donors. Planned OC also avoids problems attributed to the involvement of ‘second parties’; that is, it can allow women to control their preserved gametes without the risk that a partner may retract consent for future use, as can occur with frozen embryos” [6].

The Centers for Disease Control (CDC) and SART are the two major agencies in the United States that annually collect and report national summaries of assisted reproductive technology (ART) cycles to the public. Reports from the CDC have indicated a consistent increase in instances of oocyte banking from both autologous users and oocyte donors in the past decade [7]. Ninety-eight percent of ART cycles in the United States are subject to oversight by the CDC [7].

While the demand for egg banking is increasing, there are still unresolved concerns regarding the possible side effects of oocyte cryopreservation technology. Because of its ability to remarkably improve the effectiveness of oocyte cryopreservation, open vitrification has widely been adopted as the method of choice for oocyte cryopreservation in the United States since 2014 [5]. This brief report utilizes data from the SART and the current literature to investigate possible intrinsic elements of oocyte cryopreservation interventions that can affect the safety and effectiveness of oocyte cryopreservation using data from donor oocytes employed in ART as a representative example.

## Trend of live-birth rates

**Fig. 1:**
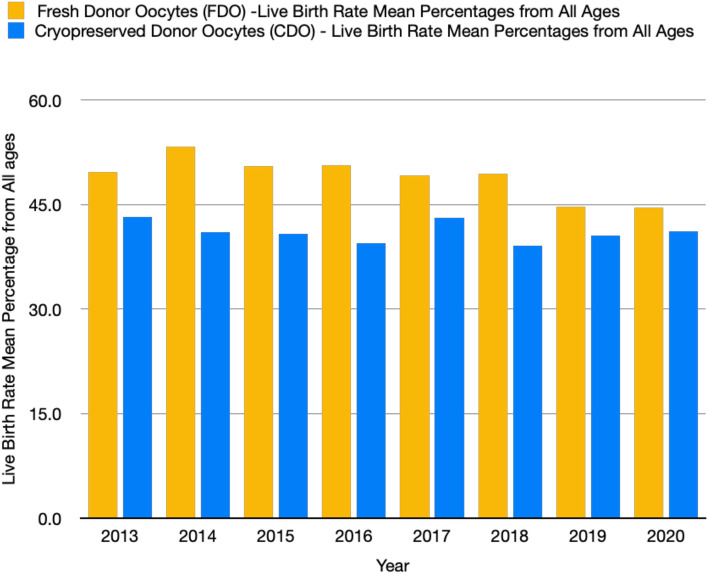
Histogram Comparing the Mean Live Birth Rate Percentages from All Ages of Fresh Donor Oocytes versus Cryopreserved Donor Oocytes from 2013–2020

Over 90% of the IVF cycles performed in United States IVF clinics have been voluntarily reported to the Society for Assisted Reproductive Technology (SART) annually since 2003. Kushnir et al*.* reported the live birth rate outcomes achieved using fresh vs. cryopreserved donor oocytes in 2013. The mean live birth rate per initiated recipient cycle from all ages of recipients was 49.6% (48.6–50.6%) with fresh donor oocytes vs. 43.2% (41.2–45.3%) with cryopreserved donor oocytes (difference, 6.4% [95% confidence interval (CI), 4.1–8.7%]; *P* < 0.001) [8]. To better understand the effects of OC on the live birth rate resulting from fresh vs. cryopreserved donor oocytes, reported data from the SART collected from 2013 to 2020 were studied, where there was at least a two-year lag time in reporting. Figure 1 shows a consistently lower mean live birth rate among recipients of all ages for cryopreserved donor oocytes when compared to fresh donor oocytes. The live birth rates resulting from a total of 34,570 fresh donor oocyte cycles from individuals of all ages were compared to a total of 22,163 cryopreserved donor oocyte cycles from individuals of all ages (Table 1). The mean of the mean live birth rate percentages from all ages reported from 2013 to 2020 were calculated and compared via independent two-tailed t test analysis using IBM SPSS Statistics. The mean of the mean live birth rate from fresh donor oocytes (FDOs) among all ages was 49.0% (44.6–53.3%) vs. 41.0% (39.1–43.2%) for cryopreserved donor oocytes (CDOs) (difference, 8.0% [95% CI, 5.35–10.57%], *p* value < 0.001 [Table 1]). This retrospective study further supported the conclusion that the primary outcome of ART, the live birth rate, is lower for CDOs than for FDOs.

Limitations related to nonadjusted patient characteristics such as recipient ages, the equalization of stimulus protocols for pregnancy, the day of embryo transfer, the number of embryos transferred, the use of a gestational carrier, infertility diagnosis, gravidity, parity, BMI, race/ethnicity, smoking status, assisted hatching, and preimplantation genetic testing (PGT-A), may have potentially led to skewed selection in this study. In addition, Acharya KS et al. [9] reported that a freeze-all strategy benefited only high responders from whom 15 or more oocytes were retrieved per cycle. Intermediate (6–14 oocytes retrieved) and low responders (1–5 oocytes retrieved) showed a lower live birth rate resulting from cryopreserved-thawed-embryo transfers compared to fresh embryo transfers. Adjustments for various types of responders were not made in this study. A further limitation of this study was the inability to discern the linkage of cycles from the same donor to different recipients because data are reported per cycle instead of per patient or per donor. Additionally, SART database collection is voluntary, and complete inclusiveness may therefore not be optimal. This study may also involve other confounding biases since information regarding the anti-Mullerian hormone levels, race and ethnicity, gravidity, parity, BMI, and smoking status of donors were not reported in the SART. Furthermore, the ages of all donors were not reported in the SART. While confounding patient and donor characteristics cannot be ignored, the large sample size in both evaluated groups (FDOs; *n* = 34,570 and CDOs; *n* = 22,163) could counterbalance these differences. This retrospective study took another look at the intrinsic effects of cryopreservation environments and cryoprotectants on the safety and effectiveness of human oocyte cryopreservation.

## Further in-depth studies of aneuploidy

The meiotic spindles of oocytes are highly sensitive to environmental changes and can be induced to depolymerize by small temperature fluctuations [10, 11]. Similar to many other publications, a recent report from the Institute of Genetics Reproduction Medical Center demonstrated an absence of differences in the aneuploidy rates of embryos derived from the in vitro fertilization of either fresh or cryopreserved donor oocytes; however, there were differences in the aneuploidy of certain chromosomes between these groups [12]. The mean ages of the donors of fresh vs. cryopreserved oocytes were 27.2 ± 2.4 and 27.6 ± 4.8 years, respectively, and this age group could be more resistant to the negative effects of cryopreservation. On chromosome 13, the nondisjunction rate was higher in embryos derived from CDOs than in those derived from FDOs (36.2% vs. 10.5%, respectively). In contrast, CDO-derived embryos showed a lower nondisjunction rate of chromosome 18 (17.2% vs. 33.1%) and the sex chromosomes (22.4% vs. 39.4%) than FDO-derived embryos [12]. These opposing changes could lead to a net zero change in the aneuploidy rate.

## Cross-omics studies of cryoprotectant effects

Based on advances in precise high-throughput genomic and cross-omics technologies, the molecular effects of a permeable cryoprotectant, dimethyl sulfoxide (DMSO), have been investigated. Caiment’s group [13] studied human 3D microtissues (MTs) in a maturing cardiac model mimicking fetal-like development and a mature hepatic model derived from human induced pluripotent stem cells (iPSCs). Under exposure to culture medium with or without 0.1% DMSO for two weeks, analyses conducted with sampling time points of 2, 8, 72, 168, 240 and 336 h revealed a clear separation of RNA samples, micro-RNA (miRNA) samples and DNA methylation patterns between untreated and 0.1% DMSO-treated 3D MTs based on principal component analysis (PCA). Distinct separation was found after as little as two hours of exposure to 0.1% DMSO. Comparisons between the two groups revealed 2051 differentially expressed genes (DEGs) in cardiac MTs and 2711 DEGs in hepatic MTs, among which 60.7% and 62.9% of the DEGs were downregulated, respectively. Through pathway analysis of DEGs, 225 significantly overrepresented pathways were identified in cardiac MTs, which corresponded to 19 clusters (among a total of 25 clusters in the Pathway Browser) and 167 pathways corresponding to 16 clusters in hepatic MTs.

The clusters that were most significantly affected by DMSO were the “metabolism” cluster (23.7% DEGs) in hepatic MTs and the “cellular responses to stress” cluster (23.5% DEGs) in cardiac MTs, although both clusters were affected in both tissue types. The “metabolic” cluster in cardiac MTs included 13.5% of the DEGs. The cardiac-specific clusters affected by DMSO were cell cycle, DNA repair, organelle biogenesis, maintenance, and chromatin organization. Although the cellular responses to stress cluster was identified for both tissue types, cellular senescence was significantly affected in cardiac MTs but was not identified in the pathway analysis of hepatic MTs.

The tissue-specific influence of DMSO was observed in the sequencing data of miRNAs, which mediate gene silencing. Among 1,105 sequenced cardiac miRNAs, 704 (= 63.7%) were differentially expressed, 59.5% which were downregulated. In hepatic MTs, among 1,033 sequenced miRNAs, 186 (= 18%) were differentially expressed, and approximately half of these miRNAs were upregulated (47.3%). The results of miRNA analysis revealed a severe deregulation of cardiac miRNA biogenesis. Although the changes in hepatic miRNA biogenesis were minimal, 18% of the identified miRNAs showed significant changes under 0.1% DMSO exposure.

*DNMT1* and *DNMT3A*, which encode epigenetic writers, were upregulated, while *TET1*, encoding an epigenetic eraser, was downregulated in cardiac MTs after 0.1% DMSO treatment. This suggested genomic hypermethylation, which could mediate reduced transcriptional activity. In contrast, in the hepatic pathway analysis, deregulation of DNA methylation mediated by *DNMT1*, *DNMT3A* and *TET1* was not observed. However, in both tissue types, transcriptional evidence of the deregulation of other related epigenetic mechanisms, such as histone methylation, was observed.

Whole-genome methylation profiling by methylated DNA immunoprecipitation sequencing (MeDIP-seq) revealed 66,178 differentially methylated regions (DMRs) in cardiac MTs but not in hepatic MTs. These alterations affected 1.1% of the covered genome, and 71% of the DMRs corresponded to a gain of methylation (46,984 hypermethylated regions vs. 19,194 hypomethylated regions). The cardiac-specific genomic DMRs suggested that cryoprotectants such as DMSO could have a more severe effect on fetal tissue development than on mature tissue development. Similar observations of more severe effects on fetal development were also found in relation to the deregulation of DNA epigenetic writers and erasers, miRNAs and RNA clustering. A decrease in ATP production was observed in 0.1% DMSO-treated samples relative to untreated samples at all sampling time points. This decrease was again more severe in fetal cardiac microtissue development [13].

Zhang et al*.* evaluated the effects of ethylene glycol (EG) as a cryoprotectant in open vitrification medium in human oocytes. Global gene expression studies based on fragments per kilobase million (FPKM) cluster analyses showed that 3740 genes were upregulated and 956 genes were downregulated in vitrified CDOs vs. FDOs [14].

## Free radical scavenger rescue

Reactive oxygen species (ROS) have been shown to be associated with abnormal development in CDOs [14]. Treatment with 10^–9^ M melatonin, a free radical scavenger, in vitrification medium used for human oocyte culture appeared to improve the adverse effects of EG. Melatonin has been shown to decrease ROS formation and revert many physiological responses of CDOs to conditions similar to those in fresh oocytes. Melatonin treatment increased the cleavage rate of CDOs to a level similar to that of FDOs in early development. However, the rate of blastocyst formation was still lower in melatonin-treated CDOs (39.02%) than in FDOs (53.49%). Live birth rates in the two groups were not reported^14^. There were insufficient data to conclude that melatonin could improve the lower live birth rate and ameliorate other adverse effects of cryopreservation in human vitrified oocytes.

## Conclusions

The growing trend of human oocyte cryopreservation is evident from data reported by the SART (Table 1). The lower live birth rate observed from CDOs compared to FDOs has been consistent across the years with relevant records (Fig. 1 and Table 1). While no increase in the aneuploidy rate has been reported for CDOs compared to FDOs, there are differences in the nondisjunction separation rate among different chromosomes [12]. Open vitrification medium can contain 15% DMSO in addition to 15% EG [1]. The adverse effects of DMSO and EG as cryoprotectants in vitrification culture medium may need to be reconsidered, since 0.1% DMSO and 10% EG have been shown to be biologically active, giving rise to dysregulated gene expression, disruption of epigenetic markers, accumulation of reactive oxygen species, and abnormal oocyte functions [13, 14]. These factors that affect live birth rates, birth defects, birth weights and developmental health cannot be ignored and must continue to be studied and followed when considering the true effectiveness of human oocyte cryopreservation.

## Data Availability

The data sets used and/or analyzed during the current study are available from the corresponding author upon reasonable request. The original reported data on live birth rates per initiated recipient cycle and oocyte banking instances can be found at https://www.sartcorsonline.com

## Abbreviations

OC: Oocyte cryopreservation
ASRM: American Society for Reproductive Medicine
FDOs: Fresh donor oocytes
CDOs: Cryopreserved donor oocytes
CI: Confidence Interval
DMSO: Dimethyl sulfoxide
EG: Ethylene glycol
IVF: In vitro fertilization
iPSCs: Induced pluripotent stem cells
3D: 3D
MTs: Microtissues
miRNA: MicroRNA
PCA: Principal component analysis
DEGs: Differentially expressed genes
MeDIP-seq: Methylated DNA immunoprecipitation sequencing
DMRs: Differentially methylated regions
FPKM: Fragments per kilobase million
ROS: Reactive oxygen species
